# TGF-beta receptor 2 downregulation in tumour-associated stroma worsens prognosis and high-grade tumours show more tumour-associated macrophages and lower TGF-beta1 expression in colon carcinoma: a retrospective study

**DOI:** 10.1186/1471-2407-7-156

**Published:** 2007-08-10

**Authors:** David Bacman, Susanne Merkel, Roland Croner, Thomas Papadopoulos, Wolfgang Brueckl, Arno Dimmler

**Affiliations:** 1 Institute of Pathology, University of Erlangen-Nuremberg, Germany; 2 Department of Surgery, University of Erlangen-Nuremberg, Germany; 3 Department of Internal Medicine I, University of Erlangen-Nuremberg, Germany; 4 Institute of Pathology, St. Vincentius hospital, Karlsruhe, Germany

## Abstract

**Background:**

Histological phenotype and clinical behaviour of malignant tumours are not only dependent on alterations in the epithelial cell compartment, but are affected by their interaction with inflammatory cells and tumour-associated stroma. Studies in animal models have shown influence of tumour-associated macrophages (TAM) on histological grade of differentiation in colon carcinoma. Disruption of transforming growth factor beta (TGF-beta) signalling in tumour cells is related to more aggressive clinical behaviour. Expression data of components of this pathway in tumour-associated stroma is limited.

**Methods:**

Tissue micro arrays of 310 colon carcinomas from curatively resected patients in UICC stage II and III were established. In a first step we quantified amount of CD68 positive TAMs and expression of components of TGF-beta signalling (TGF-beta1, TGF-beta receptors type 1 and 2, Smad 3 and 4) in tumour and associated stroma. Further we analyzed correlation to histological and clinical parameters (histological grade of differentiation (low-grade (i.e. grade 1 and 2) vs. high-grade (i.e. grade 3 and 4)), lymph node metastasis, distant metastasis, 5 year cancer related survival) using Chi-square or Fisher's exact test, when appropriate, to compare frequencies, Kaplan-Meier method to calculate 5-year rates of distant metastases and cancer-related survival and log rank test to compare the rates of distant metastases and survival. To identify independent prognostic factors Cox regression analysis including lymph node status and grading was performed.

**Results:**

High-grade tumours and those with lymph node metastases showed higher rates of TAMs and lower expression of TGF-beta1. Loss of nuclear Smad4 expression in tumor was associated with presence of lymph node metastasis, but no influence on prognosis could be demonstrated. Decrease of both TGF-beta receptors in tumour-associated stroma was associated with increased lymph node metastasis and shorter survival. Stromal TGF-beta receptor 2 expression was an independent prognostic factor for cancer related survival.

**Conclusion:**

Histological phenotype and clinical behaviour of colon cancer is not only influenced by mutational incidents in tumour cells but also affected by interaction of tumour tissue with inflammatory cells like macrophages and associated stroma and TGF-beta signalling is one important part of this crosstalk. Further studies are needed to elucidate the exact mechanisms.

## Background

Tumours do not exclusively consist of neoplastic epithelial cells, but are also accompanied by a stromal compartment composed of a variety of non-malignant cells, such as fibroblasts, inflammatory cells, and endothelial cells, as well as extracellular elements [1,2] Nonetheless in the past cancer research has been focused primarily on oncogenic events in tumour cells. It has, however, become increasingly clear that the tumour environment plays an important role in malignant disease, and a correlation between (chronic) inflammation and human predisposition to carcinogenesis has been demonstrated in several malignancies [3-5].

The majority of leukocytes that infiltrate the neoplastic stroma consist of macrophages, which are referred to as tumour-associated macrophages (TAMs)[1,4,6]. Clinical observations have shown that the presence of abundant TAMs can be associated with malignant behaviour in breast, prostatic, ovarian, and cervical carcinomas [4]. For other types of cancer, such as gastric, lung, and colorectal carcinomas, opposing data have been reported[4,7-9]. Thus, the biological significance and possible clinical implications of TAMs' presence are not yet fully understood.

Maintenance of epithelial tissues needs the stroma. When the epithelium changes, the stroma inevitably follows. Crosstalk between tumour and stromal compartment is based on several signalling pathways. One important cytokine in this context is transforming growth factor beta (TGF-β). The TGF-β superfamily of secreted polypeptides consists of three 25 kDa-proteins (TGF-β1, 2 and 3) and regulates cell proliferation, differentiation, motility, apoptosis and extracellular matrix formation in a variety of different cell types [10-12]. TGF-β serves as a tumour suppressor pathway in the normal colon by inhibiting cell proliferation and inducing apoptosis [13-15]. During late stages of colorectal carcinogenesis, TGF-β serves as a tumour promoter [16,17] and is often over expressed. A high expression level of TGF-β in the primary tumour is associated with advanced stages[18], tumour recurrence [19], and decreased survival[18].

The TGF-β signal is transduced by a pair of transmembrane serine-threonine kinase receptors[11]. TGF-β binds primarily to TGF-β-R2 receptor homodimers, which then form heterotetrameric complexes with two TGF-β-R1 molecules. As a consequence, the TGF-β-R2 kinase phosphorylates TGF-β-R1, thereby activating its serine-threonine kinase. In response to receptor activation, two cytosolic proteins, Smad2 and Smad3, become transiently associated with and phosphorylated by the TGF-β-R1 kinase. After their activation, Smad2 and Smad3 form heteromeric complexes with a third homologue, Smad4. These complexes are translocated to the nucleus, bind to DNA in a sequence-specific manner, and regulate gene transcription[11]. The resulting repression of c-*myc *and induction of cyclin-dependent kinase inhibitors as well as cdc25A phosphatase lead to G_1 _phase cell cycle arrest.

Most colorectal cancers escape the tumour suppressor effects of TGF-β as demonstrated by their resistance to the antiproliferative and apoptotic effects of TGF-β [16,17]. The molecular mechanisms by which colorectal cancers escape the tumour suppressor effects of TGF-β are an area of active investigation. A subset of colorectal cancers has been shown to have mutations or down-regulation of the type 1 receptor [20], type 2 receptor [21], Smad2 [22,23] and Smad4 [24-26].

Most studies so far focussed on alterations of the TGF-β pathway in the tumour cells. In the present study we investigated in addition to tumour-associated macrophages alterations of this signalling in tumour-associated desmoplastic stroma and their relation to histological tumour grade, regional and distant metastasis rates and survival in a group of colon carcinoma patients that underwent colon resection.

## Methods

### Tumour samples

Cases of colon adenocarcinomas were retrieved retrospectively from the files of the Department of Pathology at the University of Erlangen-Nuremberg, Germany. The specimen had been formalin-fixed, paraffin-embedded and tumour diagnosis made on haematoxylin and eosin (HE) sections. In the present study 310 tumour samples of patients were included, which underwent radical resection with formal regional lymph node dissection between 1991 and 2001 and received complete surgical resection of their colon carcinoma on clinical and pathohistological examination. Only solitary colon carcinomas except appendix tumours were included. Further exclusion criteria were: anamnestic or synchronous other malignant tumours, known familial adenomatous polyposis, colitis ulcerosa or Crohn's disease, neoadjuvant therapy, synchronous distant metastases, emergency operation, perioperative death and unknown tumour stage at the end of follow up. Clinical data is summarized in table 1. The study was carried out in compliance with the Helsinki Declaration. Ethics Committee, Faculty of Medicine, University of Erlangen-Nuremberg approved research on anonymized archived tumor material of patients in a general statement from January 24^th^, 2005.

**Table 1:** Clinical data of included colon carcinoma patients

patient data	
number of patients	n = 310
of these: male	n = 189 (61%)
female	n = 121 (39%)
median age	64 y (range 28–91 y)
median follow up	91 mo (range 1–177 mo)
adjuvant chemotherapy	n = 48 (16%)

tumour site	

caecum	n = 31 (10.0%)
colon ascendens	n = 51 (16.5%)
flexura hepatica	n = 21 (6.8%)
colon transversum	n = 32 (10.3%)
flexura lienalis	n = 15 (4.8%)
colon descendens	n = 14 (4.5%)
colon sigmoideum	n = 146 (47.1%)

tumour stage (UICC) or lymph node metastasis	

II or N0	n = 178 (57.4%)
III or N+	n = 132 (42.6%)

grading	

low-grade (G1 and G2)	n = 257 (82.9%)
high-grade (G3 and G4)	n = 53 (17.1%)

depth of invasion	

pT2	n = 24 (7.7%)
pT3	n = 255 (82.3%)
pT4	n = 31 (10.0%)

distant metastasis after 5 years follow up	n = 62 (20.0%)

### Tissue micro array technique

A map of the receiver blocks was prepared with coordinates for each sample to correctly identify the tumour samples. Under a microscope nonnecrotic carcinoma areas and surrounding areas of immediately adjacent desmoplastic stroma were marked with an indelible pen on the HE whole section of each donor block.

The tissue micro arrayer (Beecher Instruments, Woodland, USA) was used as follows: cores of 0.6 mm diameter were punched from the donor blocks and positioned in a recipient paraffin array block in smaller holes of 0.4 mm for best adhesion of the samples to the array block. Three cores of tonsil tissue were positioned on the upper left corner of each recipient block for correct orientation on the array of the recipient block. The array blocks were then incubated for 30 min at 37°C to improve adhesion between cores and paraffin of the recipient block. Afterwards the blocks were cut with a standard microtome (Microm, Heidelberg, Germany).

### Immunohistochemistry

**Figure 1:**
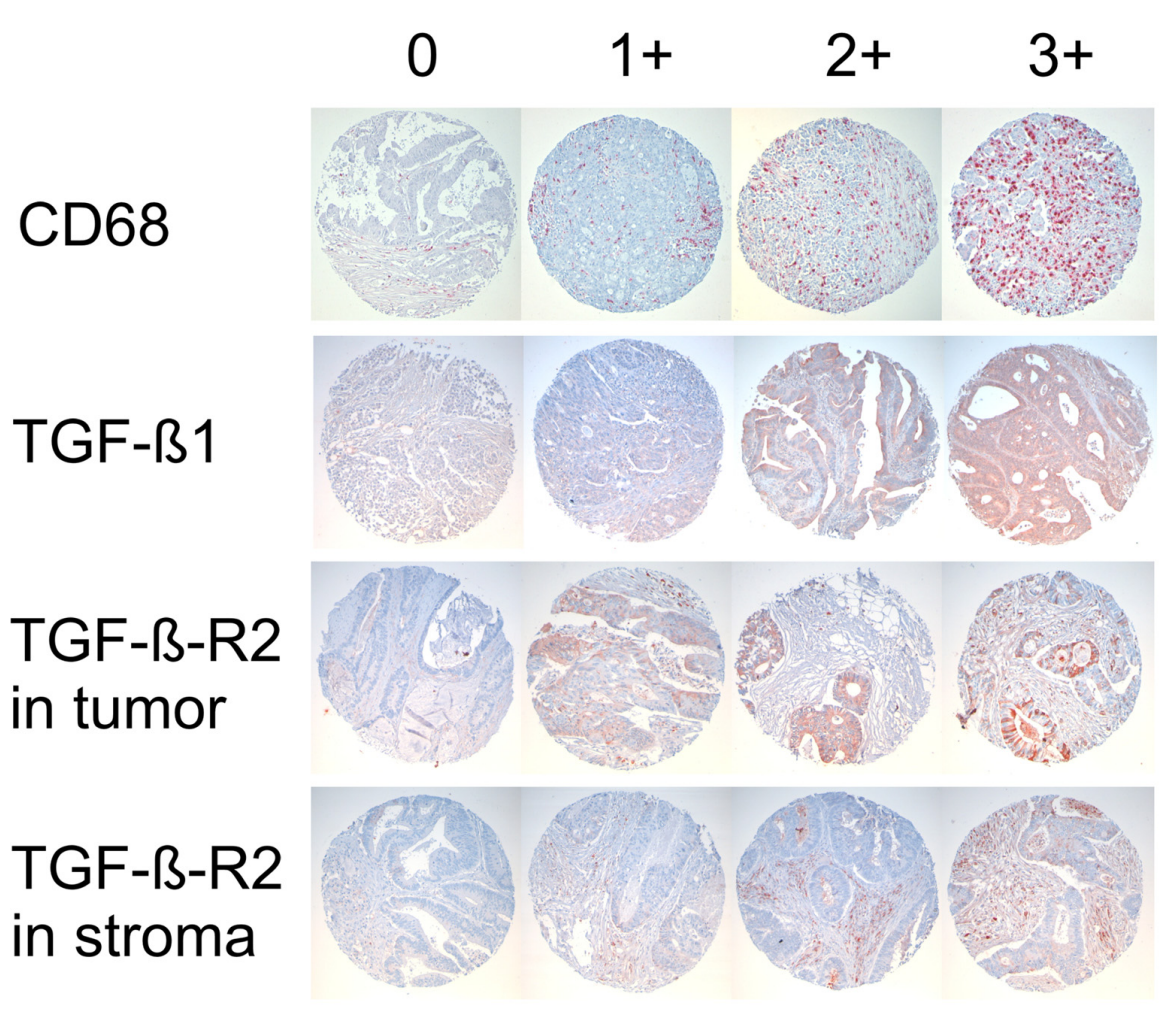
**Immunohistochemical analysis of tumor and associated stroma**. Examples for degree of TAM infiltration in CD68 staining and levels of expression of TGF-β1 and TGF-β-R2 in tumour and associated stroma. Note change of differentiation grade with increase in TAMs.

Sections of 5 μm from the fourteen tissue arrays were cut onto silane-treated Super Frost slides (CML, Nemours, France) and left to dry overnight. The slides were deparaffinized in xylene and rehydrated in pure ethanol. Endogenous peroxidase was blocked using 3 % hydrogen peroxide in methanol for 30 min. The slides were then placed in a microwave oven in citrate buffer (TGF-β1, TGF-β-R1, TGF-β-R2, Smad3, CD68) for 45 min at 120 to 85°C for antigen retrieval with a subsequent biotin-streptavidin-peroxidase detection technique or in TRS6 (DAKO, Hamburg, Germany) buffer (Smad4) at the same processing conditions with subsequent tyramide signal amplification coupled alkaline phosphatase (DAKO, Hamburg, Germany) -Fast Red (Sigma-Aldrich, Munich, Germany) detection technique. All slides were then processed manually. Antibodies to TGF-β1 (mouse anti-human monoclonal, clone TGFB17, dilution 1:20, Novo Castra, Newcastle upon Tyne, Great Britain), TGF-β-R1 (mouse anti-human monoclonal, clone 8A11, dilution 1:100, Novo Castra, Newcastle upon Tyne, Great Britain), TGF-β-R2 (goat anti-human polyclonal, AF-241-NA, dilution 1:100, Wiesbaden, Germany), Smad3 (rabbit anti-human polyclonal, 51–500, dilution 1:200, Zymed, San Francisco, USA), Smad4 (mouse anti-human monoclonal, sc-7966, dilution 1:50, Santa Cruz Biotechnology, Heidelberg, Germany), CD68 (mouse anti-human monoclonal, clone PG-M1, dilution 1:200, DAKO, Hamburg, Germany) were used. The slides were counterstained with Mayer's haemalaun. To exclude non-specific reactions of secondary antibodies or the different detection systems control specimen were processed without primary antibodies. In the CD68 staining four categories of macrophage infiltration of the tumour tissue were assessed (none (0), mild (1), intermediate (2), strong (3), see fig. 1). In the TGF-β1 staining four degrees of cytoplasmatic staining intensity (none (0), mild (1), medium(2), strong (3), fig. 1) in tumour and surrounding stroma were assessed separately. For the TGF-β-R1 and TGF-β-R2 membranous staining the same four categories were applied (fig. 1). Presence or absence of nuclear and cytoplasmatic staining was assessed for Smad 3 and 4 in tumour and surrounding stroma (for examples [see Additional file 1]).

### Statistical analysis

The Kaplan-Meier method was used to calculate 5-year rates of distant metastases and cancer-related survival. The log rank test was used to compare the rates of distant metastases and survival. To identify independent prognostic factors Cox regression analysis including lymph node status and grading was performed. To compare frequencies Chi-square test, or Fisher's exact test when appropriate, was used. A p-value of less than 0.05 was considered to be significant. All analyses were performed using the statistical software SPSS for Windows Version 13 (SPSS Inc., Chicago, USA).

## Results

The results are summarized in table 2.

**Table 2:** Content of tumour-associated macrophages and expression of TGF-β components in tumour and -associated stroma

tumour	TAMs			TGF-β1			TGF-β-R1			TGF-β-R2			Smad3			Smad4		
n	low (0–1)	high (2–3)	p value	low (0–1)	high (2–3)	p value	low (0–1)	high (2–3)	p value	neg.	pos.	p value	neg.	pos.	p value	neg.	pos.	p value
low-grade	248	7	**<0.001**	165	90	**0.009**	239	16	0.148	245	10	0.278	111	146	0.907	39	214	0.295
high-grade	39	13		44	9		47	7		50	4		22	30		5	47	
N0	167	9	**0.019**	113	63	0.202	163	14	0.581	167	10	0.630	79	99	0.579	35	139	**0.004**
N+	120	11		96	36		123	9		128	4		54	77		9	122	
5 ycrs	87.0	80.0	0.261	85.6	88.8	0.459	86.4	87.0	0.787	87.0	78.6	0.631	86.1	86.6	0.991	92.5	85.4	0.896
5 ydm	19.4	30.0	0.241	18.7	22.6	0.463	19.9	17.4	0.851	19.9	21.4	0.701	19.5	19.9	0.795	17.3	20.7	0.419

stroma	TAMs			TGF-β1			TGF-β-R1			TGF-β-R2			Smad3			Smad4		
n	low (0–1)	high (2–3)	p value	low (0–1)	high (2–3)	p value	low (0–1)	high (2–3)	p value	neg.	pos.	p value	neg.	pos.	p value	neg.	pos.	p value

low-grade	152	104	0.134	256	0	n.a.	162	94	1.000	98	158	0.229	140	117	0.653	7	249	1.000
high-grade	26	28		52	0		34	20		25	28		27	26		1	52	
N0	59	39	0.141	176	0	n.a.	102	76	**0.033**	56	121	**0.001**	98	80	0.627	5	172	1.000
N+	119	93		132	0		94	38		67	65		69	63		3	129	
5 ycrs	84.1	87.8	0.897	86.4	n.a.	n.a.	84.7	89.5	0.245	82.3	89.3	**0.003**	87.5	85.4	0.753	83.3	86.5	0.812
5 ydm	12.6	20.0	0.643	20.7	n.a.	n.a.	24.5	12.3	0.072	25.4	16.6	0.087	21.0	18.9	0.392	14.3	20.2	0.582

5 ycr: 5 year cancer related survival (%); 5 ydm: 5 year rate of distant metastasis (%); n.a.: not available.

### TAMs

Macrophages in tumour-associated stroma were more abundant than those infiltrating tumour tissue (mean score 1.8 vs. 0.5). We found significantly higher levels of tumour-associated macrophages in high-grade tumours (p < 0.001) and tumours with lymph node metastases (p = 0.019). 58% (167 of 287) of tumours with no or only mild infiltration by TAMs compared to 45% (9 of 20) of tumours showing intermediate and strong infiltration had no lymph node metastases. There were no significant differences in 5-year survival (87% vs. 80%) or distant metastases (19.4% vs. 30.0%) between the two groups (p = 0.261 and p = 0.241 respectively).

### Components of TGF-β pathway in tumour tissue

In 296 of 308 tumours (96%) expression of TGF-β1 could be demonstrated. Low-grade tumours showed higher TGF-β1 expression (p = 0.009; mean expression level 1.40 for low-grade vs. 0.91 for high-grade tumours). No correlation between TGF-β1 expression and lymph node metastasis, cancer related survival or distant metastasis could be demonstrated.

Only few tumours showed expression of TGF-β receptors (27% or 84 of 309 for type1; 5% or 14 of 295 for type 2). The rate of receptor expression was not linked to histological grade, regional or distant metastasis or survival.

In the majority of tumours (nuclear or cytoplasmatic) expression of Smad3 (91%; 279 of 309 tumours) and Smad4 (100% or all 305 tumours) was found. Most tumours showed nuclear expression of Smad3 (57%; 176 of 309) and Smad4 (86%; 261 of 305), but no correlation to histological grade could be demonstrated. More nodal positive tumours (93% or 122 of 131) than cases without lymph node metastasis (80% or 139 of 174) showed nuclear Smad4 expression (p = 0.004). No correlation was found between expression of Smads and distant metastasis or survival.

### Components of TGF-β pathway in tumour-associated stroma

Expression of TGF-β1 in tumour-associated stroma could not be demonstrated. Expression of TGF-β receptors and Smads in stroma was restricted to spindled and sometimes stellar stromal cells apparently representing fibroblasts and myofibroblasts. In most cases TGF-β receptors were expressed in the tumour-associated stroma (60% or 188 of 310 for type 1; 60% or 186 of 309 for type 2). For both receptors no correlation to histological grade was found. Higher TGF-β receptor expression in stroma was associated with significant decrease of nodal positive tumours (table 2, p = 0.033 for type 1 and p = 0.001 for type 2). This was partially reflected by significantly better 5-year cancer related survival for receptor positive compared to negative cases in the nodal positive group (type 1: 86% vs. 69%; p = 0.011 and type 2: 83% vs.76%; p = 0.032). TGF-β receptor expressing cases showed a trend towards lower rates of distant metastasis, although this difference was not significant (table 2; 24.5% vs. 12.3%; p = 0.072 for type 1 and 25.4% vs. 16.6%, p = 0.087 for type 2).

Nuclear expression in the stroma was detected in 46% (143 of 310) of tumours for Smad3 and 97% (301 of 309) for Smad4. No correlation to histological grade, local and distant metastasis or survival could be demonstrated.

### Multivariate analysis

**Figure 2:**
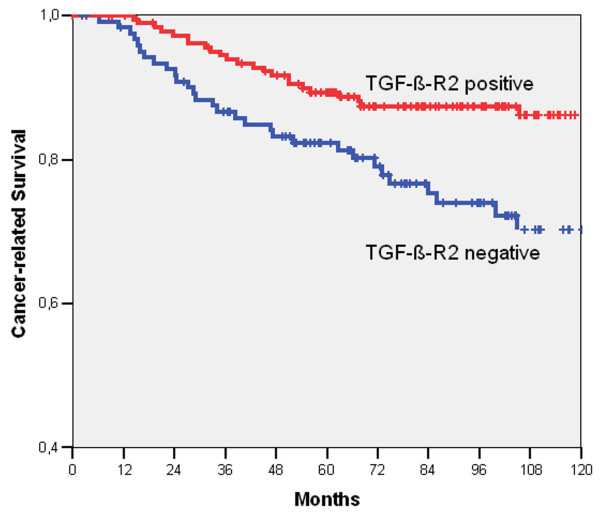
**Kaplan-Meier survival curve for TGF-β-R2 expression in tumor-associated stroma**. Cancer-related survival of colon carcinoma patients according to TGF-β-R2 expression in tumour-associated stroma.

In univariate analysis using log rank test presence of lymph node metastasis (identical to tumour stage: 5-year cancer-related survival rate: N0 or II: 92.1% vs. N+ or III: 79.5%, p <0,001, table 3), vessel infiltration (V0: 89.0% vs. V1: 58.3%, p < 0,001) and TGF-β-R2 expression in tumour-associated stroma (negative: 82.3% vs. positive: 89.3%, p = 0,003) showed statistically significant correlation to cancer-related survival, whereas for all other factors no association could be demonstrated (see table 3). On multivariate analysis using Cox regression analysis besides vessel infiltration and lymph node status expression of TGF-β-R2 in the stroma (RR 2.2, 95%CI 1.2–3.8, p = 0.007, table 3) was an independent prognostic factor for cancer-related survival as reflected in Kaplan-Meier survival curve (see Fig. 2).

**Table 3:** Results of univariate and multivariate analysis

	univariate analysis				
	score	n	5 year survival rate	SE	p value

pT	2	24	100		0.226
	3	255	85.6	2.2	
	4	31	83.0	6.9	
grading	low-grade	257	86.9	2.2	0.446
	high-grade	53	84.8	5	
lymph node status	N0 or II	178	92.1	2.1	<0.001
or stage	N+ or III	132	79.5	3.5	
vessel infiltration	V0	285	89.0	1.9	<0.001
	V1	24	58.3	10.1	
chemotherapy	applied	261	87.1	2.1	0.700
	not applied	49	83.5	5.3	
TGF-β1 in tumour	low (0–1)	209	85.5	2.5	0.462
	high (2–3)	99	88.6	3.2	
Smad3 in tumour	negative	133	88.1	2.9	0.941
	positive	176	85.3	2.7	
Smad4 in tumour	negative	44	92.5	4.2	0.896
	positive	261	85.4	2.2	
TGF-β-R1 in stroma	negative	122	83.8	3.4	0.121
	positive	188	88.3	2.4	
TGF-β R2 in stroma	negative	123	82.3	3.5	0.003
	positive	186	89.3	2.3	

	multivariate analysis				

	score	n	relative risk RR	95% confidence intervall	p value

TGF-β R2 in stroma	negative	122	1		
	positive	186	2.2	1.2–3.8	0.007
lymph node status	N0	177	1		
	N+	131	2.1	1.2–3.8	0.012
vessel infiltration	V0	284	1		
	V1	24	4.1	2.1–7.9	<0.001

## Discussion

Macrophage functions are profoundly affected by micro-environmental signals and can range from powerful induction of inflammatory responses to immunosuppression [27]. Although it has been demonstrated previously that macrophages are capable of killing tumour cells *in vitro *and *in vivo*, evidence is now emerging in support of a tumour-promoting role for macrophages, and it has recently been hypothesized that macrophages, residing within neoplastic tissues, are educated by tumour cells to perform auxiliary oncogenic functions such as matrix break-down and induction of angiogenesis [27-30]. In the present study we found higher levels of TAMs in high-grade tumours and tumours with lymph node metastasis. Although there was a trend towards lower rates of distant metastasis and better survival rates in TAM rich tumours, this difference was not statistically significant. As this group accounts for less than 7% of tumours in our study this result might not be reproducible in larger series. Nonetheless several observations could explain these apparently contradictory effects of macrophages in disease outcome. In a recent experimental study the level of macrophage accumulation was found to correlate with macrophage functionality: high-level secretion of monocyte chemoattractant protein-1 (MCP-1) resulted in massive infiltration of macrophages with subsequent tumour regression. By contrast, low-level secretion of MCP-1 led to moderate infiltration of macrophages and melanoma progression [31]. Second, macrophage activity may alter over time as a consequence of tumour-induced immune dysfunction, since nitric oxide production, which is a major effector molecule for tumour cell killing, was suppressed in macrophages of tumour-bearing mice [32]. Third, a conceptual framework referred to as the so called macrophage balance hypothesis has been proposed, defining two different macrophage populations ranging from polarized potent killer/effector M1 cells to alternatively activated M2 macrophages with tumour-promoting characteristics. In a recently published study, the presence of macrophages directed tumours towards a histologically more malignant phenotype characterized by extensive stromal reaction, disorganized matrix deposition, and neovascularization [33]. Nonetheless, depletion of macrophages deprived tumour inhibitory functions as well, resulting in enhanced growth and decreased survival, emphasizing the complexity of the proposed macrophage balance. We think balance between the following actions of tumour-associated macrophages could account for some observations in the present study: At the primary tumour site macrophage dependent and cytokine mediated promoting activity might increase local infiltrative growth, enhancement of lymphangiogenesis and lymphatic vessel infiltration. On the other hand tumouricidal activity of macrophages might be restricted to single and isolated tumour cells on invasion front and founder cells of early hematogenic metastases. Reducing the number of macrophages per se, which has been proposed as a therapeutic strategy, may therefore not constitute the most optimal approach. However, specifically counteracting tumour-promoting characteristics and/or enhancing macrophage tumouricidal activity might be promising for future therapies. The second question we addressed in this study was whether alterations in TGF-β signalling are related to histological tumour grade and clinical behaviour of colon cancers. First we analyzed important factors of TGF-β signalling pathway in the epithelial component of the tumours. Immunostainable TGF-β1 has been detected in the malignant epithelial cells in the majority of our investigated cases of colon carcinomas and expression was shown to be higher in low-grade tumours, as shown recently [34], whereas we found no correlation to lymph node metastases, tumour stage or survival, although there was a non-significant trend towards lower TGF-β1 expression in tumours with regional lymph node metastases. This is in contrast to studies, where TGF-β1 overexpression in advanced stages of colorectal cancers has been reported and the intensity of the staining seems to correlate with advancing stages of tumour progression[35,36]. However, interpretation of these studies is complicated by difficulties associated with distinguishing the biologically inactive, latent form of TGF-β1 from its activated form.

Most tumours in the present study showed no detectable TGF-β receptor expression. The loss of TGF-β receptor expression in colon cancer either through mutation or downregulation has been reported [20,21], but like in our study no correlation to histological grade or clinical outcome was found[37]. In contrast to loss of TGF-β receptors in tumour tissue we found frequent activation of Smad signalling indicated by nuclear expression of Smad3 and Smad4 in tumour and surrounding stroma, but only nuclear expression of Smad4 in the malignant epithelial component was correlated to presence of lymph node metastasis. Although experimental data suggest tumour suppressive effects of functionally active Smad4[38,39] and loss of Smad4 in colorectal cancer is associated with advanced stage disease, presence of lymph node metastasis and poor prognosis [40-43], others reported retained Smad4 expression in high-grade colorectal carcinoma and suggested loss of Smad4 is a late event in colorectal carcinogenesis [44]. In our study no influence of reduced Smad4 expression on prognosis was found. A possible explanation could be that in our study nodal positive cases (i.e. stage III patients) had an excellent 5 year survival of almost 80% indicating good local tumor control. The reduction of local recurrences possibly through extended lymphadenectomy could eliminate the prognostic influence of reduced Smad4. Activation of Smad signalling in our cases as indicated by frequent nuclear expression in the absence of TGF-β-R expression might occur primarily through TGF-β independent mechanisms, e.g. activin signalling [45]. Most authors so far focused primarily on the malignant epithelial component in tumours, so expression data of components of TGF-β pathway in tumour-associated stroma is rare. Unlike others [21] we found no expression of TGF-β1 in stroma. In our study frequent nuclear expression of Smads showed no correlation to tumour grade or clinical outcome. The most important finding in our study was an association of expression of both TGF-β receptors to presence of regional metastases. In patients with regional metastasized tumours decreased receptor expression in tumour-associated stroma was associated with shorter survival, whereas incidence of distant metastases showed only minor non-significant increase. Most importantly besides the well-established influence of lymph node metastasis and vessel infiltration on cancer-related survival in our study we were able to show that TGF-β-R2 expression in the stroma is an independent prognostic factor. Main effector cells for TGF-β1 in tumour-associated stroma are fibroblasts and myofibroblasts and decrease of TGF-β receptor expression in stroma as in epithelial tumour tissue might occur via mutation or downregulation [20,21] and reflects functional alteration of TGF-β signalling. Paracrine effects of TGF-β in the stroma can be summarized as locally tumour promoting and implicate stimulation of angiogenesis, escape from immunosurveillance and recruitment of myofibroblasts (as reviewed in [46]). As in models of tumour escape from chemotherapy[47], disruption of TGF-β signalling in the stroma via decreased receptor expression might drive tumour evolution towards a prometastatic phenotype. This might account for decreased survival, although other mechanisms, e.g. reduction of indirect antiproliferative TGF-β feedback effects from stroma to tumour, are conceivable.

## Conclusion

In summary not only mutational incidents in tumour cells but also interaction of tumour tissue with inflammatory cells like macrophages and associated stroma through TGF-β signalling modulates histological phenotype and clinical progression in colorectal cancer. Further studies are needed to clarify the underlying mechanisms.

## Competing interests

The author(s) declare that they have no competing interests.

## Authors' contributions

DB carried out assembly of tissue micro arrays and immunohistochemical stainings including evaluation and was involved in preparation of the manuscript. RC participated in the design of the study and was responsible for collection of clinical data. TP participated in the design of the study and coordination and helped to draft the manuscript. SM participated in the design of the study and performed the statistical analysis. WB participated in the design of the study and selection of patients. AD conceived of the study, and participated in its design and coordination and helped to draft the manuscript. All authors read and approved the final manuscript.

## Pre-publication history

The pre-publication history for this paper can be accessed here:



## Supplementary Material

Additional file 1 Smad3 and Smad4 expression in tumour. The figure shows examples of immunohistochemical stainings of Smad3 and Smad 4 in tissue micro arrays. Tumours with loss of Smad4 expression were not found. Click here for file
