# Supplementary figures and images for: TGF-beta receptor 2 downregulation in tumour-associated stroma worsens prognosis and high-grade tumours show more tumour-associated macrophages and lower TGF-beta1 expression in colon carcinoma: a retrospective study
Source: BMC Cancer. 2007 Aug 10;7:156. doi: 10.1186/1471-2407-7-156 (PMC1988827; doi:10.1186/1471-2407-7-156)

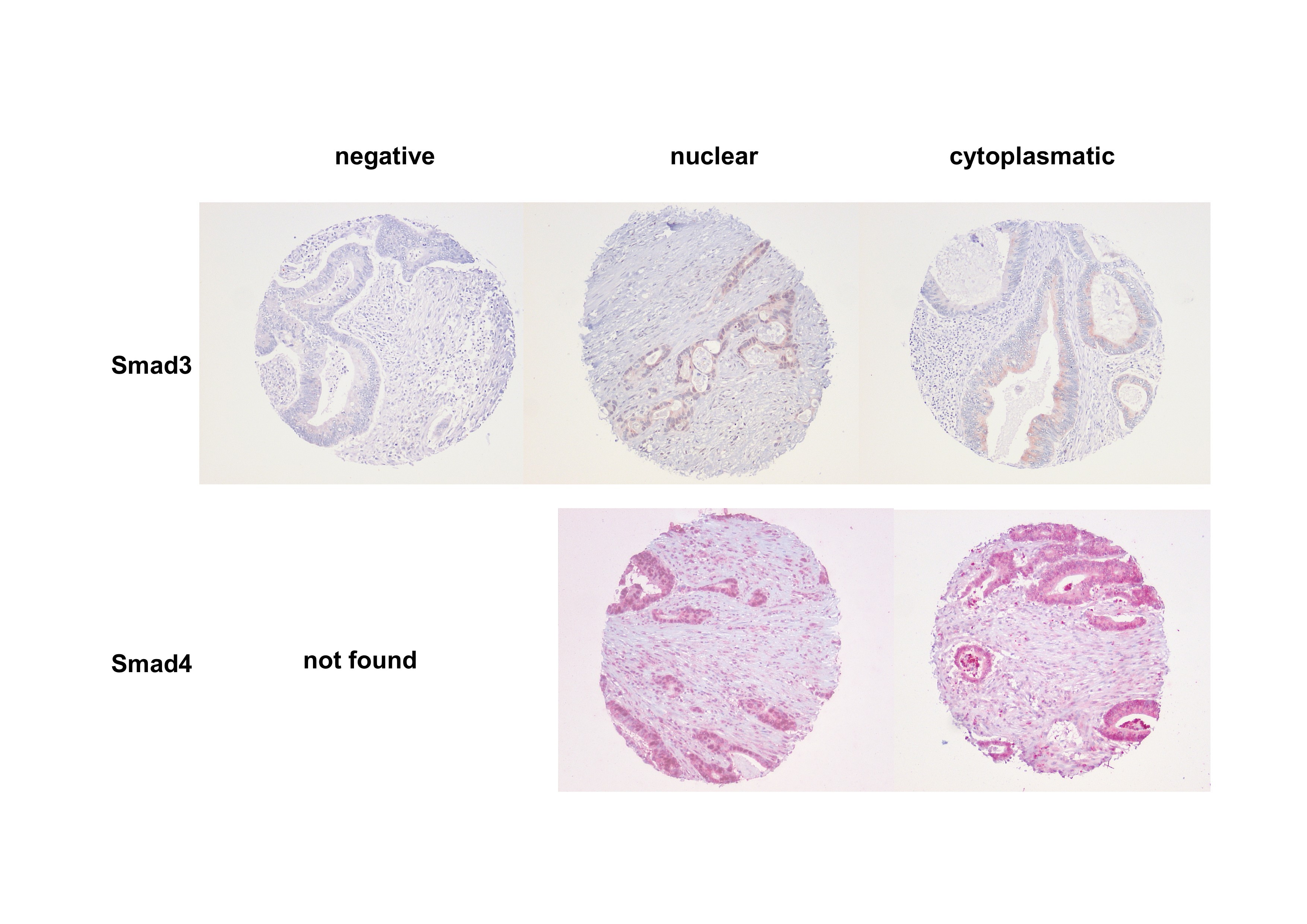

Supplement: Additional file 1 — Smad3 and Smad4 expression in tumour. The figure shows examples of immunohistochemical stainings of Smad3 and Smad 4 in tissue micro arrays. Tumours with loss of Smad4 expression were not found. [file 1471-2407-7-156-S1.jpeg]
